# Phytoremediation of Cadmium: Physiological, Biochemical, and Molecular Mechanisms

**DOI:** 10.3390/biology9070177

**Published:** 2020-07-21

**Authors:** Ali Raza, Madiha Habib, Shiva Najafi Kakavand, Zainab Zahid, Noreen Zahra, Rahat Sharif, Mirza Hasanuzzaman

**Affiliations:** 1 Key Lab of Biology and Genetic Improvement of Oil Crops, Oil Crops Research Institute, Chinese Academy of Agricultural Sciences (CAAS), Wuhan 430062, China; alirazamughal143@gmail.com; 2 Centre of Agricultural Biochemistry and Biotechnology (CABB), University of Agriculture, Faisalabad 38040, Pakistan; madihahabib217@gmail.com; 3 Laboratory of Plant Physiology, Department of Biology, Faculty of Science, Razi University, Kermanshah 6714115111, Iran; shiva.najafik@gmail.com; 4 Institute of Environmental Sciences and Engineering (IESE), School of Civil and Environmental Engineering (SCEE), National University of Sciences and Technology (NUST), Islamabad 44000, Pakistan; zainabzahid5@gmail.com; 5 Department of Botany, University of Agriculture, Faisalabad 38040, Pakistan; noreenzahra59@gmail.com; 6 College of Horticulture, Northwest A&F University, Yangling 712100, China; rahatsharif2016@nwafu.edu.cn; 7 Department of Agronomy, Faculty of Agriculture, Sher-e-Bangla Agricultural University, Sher-e-Bangla Nagar, Dhaka-1207, Bangladesh

**Keywords:** heavy metals, phytoremediation, antioxidant defense system, genetic engineering, microbes, metallothionein, omics, phytochelatins

## Abstract

Cadmium (Cd) is one of the most toxic metals in the environment, and has noxious effects on plant growth and production. Cd-accumulating plants showed reduced growth and productivity. Therefore, remediation of this non-essential and toxic pollutant is a prerequisite. Plant-based phytoremediation methodology is considered as one a secure, environmentally friendly, and cost-effective approach for toxic metal remediation. Phytoremediating plants transport and accumulate Cd inside their roots, shoots, leaves, and vacuoles. Phytoremediation of Cd-contaminated sites through hyperaccumulator plants proves a ground-breaking and profitable choice to combat the contaminants. Moreover, the efficiency of Cd phytoremediation and Cd bioavailability can be improved by using plant growth-promoting bacteria (PGPB). Emerging modern molecular technologies have augmented our insight into the metabolic processes involved in Cd tolerance in regular cultivated crops and hyperaccumulator plants. Plants’ development via genetic engineering tools, like enhanced metal uptake, metal transport, Cd accumulation, and the overall Cd tolerance, unlocks new directions for phytoremediation. In this review, we outline the physiological, biochemical, and molecular mechanisms involved in Cd phytoremediation. Further, a focus on the potential of omics and genetic engineering strategies has been documented for the efficient remediation of a Cd-contaminated environment.

## 1. Introduction

Cadmium (Cd) is a non-essential element for plants and humans but is present in many soils in excessive amounts [1,2]. When it enters into the food chain, it poses a major threat to the living biota. The control of Cd accumulation in plants is complicated by the fact that most of the essential nutrient transporters, such as copper (Cu), manganese (Mn), iron (Fe), and zinc (Zn), also facilitate Cd uptake [2]. Cd stress alters plant growth, as evident from a reduced dry matter yield and leaf area, and stunted growth [3,4,5,6]. Cd affects plant growth at both the morphological and physiological level [7]. At the whole plant level, Cd toxicity includes leaf chlorosis, a delay in the growth rate, and inhibition of respiration and photosynthesis [8], increased oxidative damage, and decreased nutrient uptake ability [9].

Generally, Cd occurs in sedimentary rocks (0.3 mg kg^−1^), lithosphere (0.2 mg kg^−1^), and soil (0.53 mg kg^−1^) [10]. Cd enrichment in soil occurs from both anthropogenic and natural sources [11]. Geologically weathering of rocks is the major natural source of Cd contaminants [12,13], while primary anthropogenic sources of Cd include agrochemicals, manufacturing, vehicular emission, irrigation wastewater, smelting, and mining [14,15]. Moreover, improper and uncontrolled waste disposable practices, sea spry, windblown dust, forest fires, and volcanic eruption also increase the Cd level in soil [12,13,14].

Apart from this, Cd toxicity has been reported to damage human physiology by various means, such as Cd-contaminated water and food. For example, Cd exposure influences the human male reproductive organs/system and deteriorates spermatogenesis and semen quality, especially sperm motility and hormonal synthesis/release. Based on experimental and human studies, it also impairs female reproduction and the reproductive hormonal balance and affects menstrual cycles [16,17]. In animals, experimental studies revealed that Cd and Cd compounds (referred to as Cd) by multiple routes of exposure prompt benign and malignant tumor formation at various sites in many species of experimental animals [18]. Besides, environmental Cd contact can cause pancreatic cancer in animals [19].

Efficient and economical remediation of contaminated urban and agriculture land is a pressing need for sustainable agriculture development prospects. Different methods, like biological, chemical, and physical, have been used for the remediation of heavy metal contaminants from soil. Some of them face limitations due to mechanical limitations, logistical problems, time, and cost. Soil remediation techniques include physical, chemical, and biological remediation; electrokinetics; and phytoremediation. Physical remediation includes both the soil high replacement method and thermal desorption method. In the soil replacement method, clean soil is used for partial and full replacement of contaminated soil [20,21]. Importing new soil dilutes the contaminated soil; however, this practice is only useful for small-scale severely contaminated soil.

Chemical remediation is a mechanical process used for leaching the contaminated soil by using liquids enriched with solvents, freshwater, and chelating agents [20]. Researchers found that ethylenediaminetetraacetic acid (EDTA) is an effective chelating agent for soil washing. Recent studies have shown that biosurfactants, such as sophorolipids, saponin, and rhamnolipids, can efficiently remove Cd from contaminated soils [22]. According to Juwarkar et al. [23], more than 92% of Cd was removed with 0.1% di-rhamnolipid. In chemical oxidation, oxidants, such as Fe^2+^-activated peroxymonosulfate, are used to degrade and oxidized the contaminant particles [24]. Bioremediation is the use of microorganisms, plants, and microbial or plant enzymes to treat the contaminated soil through natural biodegradation. Some Cd-removing microorganisms include *Aspergillus niger* (fungus) [25], *Pleurotus ostreatus* [26], *Spergilus versicolor* [27], *Fomitopsis pinicola*, *Pseudomonas aeruginosa*, *Streptomyces*, and *Bacillus* [28]. Electrokinetics is another technique to remediate heavy metals from soil by using an electrical current [29,30]. In this context, Shen et al. [31] reported an accumulation of 99% of total Cd at the cathode after remediating soil with approaching anode electro-kinetics. Similarly, Li et al. [32] reported a removal of 97.32% of the total Cd through electroosmotic and electromigration, which was positively correlated with the electric voltage. Recently, it has become the best technique and seems to be a promising alternative to conventional approaches.

Phytoremediation is a cost-effective and eco-friendly technique for remediating soils. Notably, phytoremediating plants uptake and accumulate Cd inside their roots, shoots, leaves, and vacuoles. Still, it takes a long time to provide fruitful results because phytoremediation is still under the investigation and progress phase, and several technical barriers have to be overcome. In the present review, we illustrate the recent advancements in the physiological, biochemical, and molecular mechanisms associated with Cd phytoremediation. Additionally, the potential of omics and genetic engineering approaches are outlined for the efficient remediation of a Cd-contaminated environment.

## 2. Plant Responses to Cadmium Toxicity

The toxic effects of Cd on plant growth and metabolism differ among plant species [3]. The Cd concentration in plants is a direct function of its presence in the soil. An increase in Cd concentration in the growth medium led to a subsequent rise in its accumulation in different parts of the plants [33,34]. It might alter the plant growth and metabolism even if present in a minute amount [33,35]. Cd application in basil seeds delayed the germination period from 4.66 to around 7–10 days [5]. Several other studies have reported high Cd accumulation in wheat seedling roots compared to shoots [36,37].

In some studies, Cd toxicity is linked with the low dry matter accumulation in roots [4], turning them black [38], and leads to a reduction in lateral root growth [39]. It has further been associated with root development with the mature apoplastic pathway, enhanced porosity, and few root tips per surface area in rice plants [40]. In the genus *Citrus*, seedlings wilted and turned yellow, followed by eventual death under cadmium chloride (CdCl_2_) treatment [41]. In contrast, tomato plants were reported to endure short-term exposure to high (250 µM) CdCl_2_ concentrations [42]. Increased exposure to Cd in carrots and radish significantly inhibited the development of radicals due to increased Cd accumulation in roots [43]. Moreover, a considerably high metal concentration was found in parsley seedlings under Cd stress, but the plants did not show any visual stress symptoms [44].

The morphological, biochemical, and physiological effects on plant growth are more pronounced with a high concentration of Cd [39]. An evaluation of the morpho-physiological growth parameters of tomatoes showed that at high Cd concentrations, the root-shoot growth decreased at a relative rate, which could be attributed to the lesser water content in the seedlings due to reduced imbibition. The literature suggests that Cd stress decreased the root-shoot length in wheat [45], peas [46], *Corchorus capsularis* [47], and *Suaeda glauca* [48]. Further, it has been documented to reduce the dry weight in wheat [49], maize [50], and tomatoes [51].

Wu et al. [52] described an increase in the net photosynthesis rate by Cd application, which translated to an increase in the net biomass. On the contrary, Cd toxicity has been reported to inhibit plant growth by decreasing the water-use efficiency (WUE) and the net rate of photosynthesis [53]. A significant reduction in the leaf area and dry mass in female *Populus cathayana* under Cd stress has been observed [54]. In contrast, growth inhibition and a disturbance in photosynthetic performance has been reported in Cd-stressed tomato [55] and cucumber [56]. Cd exposure has further been reported to decrease the stomatal conductance and net rate of photosynthesis in rapeseed [57].

Cd toxicity also affects the plasma membrane of plants, which could be attributed to electrolyte leakage [58], and membrane proteins like H-ATPase inhibition [42]. It has been known to affect the DNA repair system [59]; thus, the stability of the genomic template was distinctly reduced in *Phaseolus vulgaris* [60] and peas [61] in response to the direct application of Cd.

Reactive oxygen species (ROS) generation in response to Cd-induced oxidative stress affects the electron transport and leaks electrons to molecular oxygen [62]. Cd was found to be toxic for peanuts at a higher dosage, marked by the production and accumulation of ROS in the cytosol. Moreover, it damaged the integrity and selective transport system of plasma membranes, leading to metal transport in cells [63]. Furthermore, the overproduction of ROS in wheat seedlings upon Cd exposure, marked by an increase in the hydrogen peroxide (H_2_O_2_) content and malondialdehyde (MDA) level, was linked to genotoxicity [64]. Being sessile, plants try to elude its harmful effects by adopting various defense mechanisms, which include antioxidant activation and other mechanisms of metal homeostasis [65]. In response, plants have developed enzymatic and non-enzymatic antioxidant mechanisms. Increased activities of catalase (CAT), superoxide dismutase (SOD), ascorbate peroxidase (APX), and peroxidase (POD) were found against increased Cd stress in *Brassica juncea* [66]. In another study, the glutamate-mediated alleviation of Cd toxicity reduced ROS-induced membrane lipid peroxidation, metal uptake, and translocation to rice shoots, and improved the chlorophyll biosynthesis [67].

## 3. Phytoremediation Processes and Their Salient Features

Phytoremediation refers to the biological cleaning of the environment (soil, water, and air) by plants. Plants make a symbiotic association with microorganisms, which helps in the remediation of the soil, particularly from heavy metals and organic pollutants. Phytoremediation is generally considered as a green technology because of its excellent decontamination ability of heavy metals with a minimum influx of secondary waste to the environment. Alternatively, phytoremediation is highly acceptable among the general public due to its ease of application, low cost, and environmentally friendly nature [1,2]. However, hampered growth activities, such as reduced biomass and increased sensitivity to Cd, were observed in the plants involved in phytoremediation processes [6].

Phytoremediation involves various processes, such as phytoextraction, phytoaccumulation, phytovolatilization, phytostabilization, and phytotransformation. The phytoextraction and phytoaccumulation processes work in association. For instance, during phytoextraction, plants uptake heavy metals, such as Cd, Zn, nickel (Ni), chromium (Cr), and other minerals and nutrients from the soil. After this, these elements accumulate in the shoots and leaves with the help of the phytoaccumulation mechanism [6]. Many plants species have been reported previously for their high accumulation capacity; these are potential candidates for phytoremediation.

In Cd phytoremediation, plants are often used to absorb or translocate Cd into harvestable plant parts. Plants have evolved many diverse adaptations to maintain normal growth even under high Cd-contaminated soils, which also includes detoxification mechanisms [68]. The Cd concentration in plant parts shows the following trend: root > stem > leaves [69]. Many techniques are being used to increase the efficiency of Cd phytoremediation (Table 1).

### 3.1. Phytoextraction

This technique is used to absorb inorganic and organic contaminants through the stem and roots. Plants that are already growing in the ecosystem should be chosen for this technique. After harvest, they are exposed to another method known as composition, or burned in an incinerator [70]. Hyperaccumulator families, such as *Scrophulariaceae*, *Lamiaceae*, *Asteraceae*, *Euphorbiaceae,* and *Brassicaceae*, are essential for this technique. Moreover, some particular plant species, like *Celosia argentea* [71], *Salix mucronata* [72], *Cassia alata* [73], *Vigna unguiculata*, *Solanum melonaena*, *Momordica charantia* [74], *Nicotiana tabacum*, *Kummerowia striata* [75], and *Swietenia macrophylla* [76], may be used as potential plant choices to increase the process of Cd phytoextraction. Moreover, a sub-division of phytoextraction, known as chelate-assisted phytoextraction, is also used as a possible solution for metals that have no hyperaccumulator species. Several amino polycarboxylic acid and chelating agents have been applied to soil to increase the solubility of trace elements. For instance, EDTA-assisted phytoextraction of Cd was preferred by Farid et al. [77]. Similarly, citric acid was used as a chelating agent to increase the Cd uptake ability of jute mallow (*Corchorus olitorius*) [78].

Phytoextraction helps to reduce metalloid toxicity by improving substrate geochemistry for future colonization of native plants [79]. It is an effective, affordable, environmentally friendly, and potentially cost-effective technique for remediating soils [80]. Despite the generally agreed advantages of phytoextraction, there are some disadvantages, such as the time required for the remediation of highly contaminated soils may be decades [81], and a limitation for mine waste applications [82]. Mostly hyperaccumulator plants have developed the capacity to accumulate only one metal and may be sensitive to the presence of other elements [81].

### 3.2. Phytostabilization

There has been a progressing shift from phytoextraction to phytostabilization. Phytostabilization is the ability of plants to store and immobilized heavy metals by binding with biomolecules; this process prevents metal transport, and converts them into less toxic substances [83]. Most of the plants growing on contaminated soils are not hyperaccumulators but work as excluders. An excluder transforms the metals and metalloids into a less toxic mobile form without extracting them from the soil and accumulates these compounds in roots by absorption or precipitation within the rhizosphere [84]. Recently, promising results of *Virola surinamensis* for Cd phytostabilization have been documented [85]. Likewise, *Miscanthus* x *giganteus* [86], and oats and white mustard [87] also have phytostabilization potential for Cd. In another example, the putative role of Fe-Si-Ca, organic fertilizers, and coconut shell biochar has been reported to enhance the phytostabilization ability of *Boehmeria nivea* L. for Cd [88].

Phytostabilization is one emerging ecofriendly phytotechnology, which immobilizes the environmental toxins [89]. Roots take part in phytostabilization, so the metal availability is reduced to the plants, thus reducing the exposure to the other tropic level of the environment [90]. At the same time, the major disadvantage is the fact that pollutant remains in the soil or in the root system, generally in the rhizosphere [91].

### 3.3. Phytofiltration

Phytofiltration is categorized as rhizofiltration that includes blastofiltration (use seedlings) and caulofilteration (use of excised plant) (Table 1) [92]. Rhizofiltration is the remediation of water in which roots effectively absorb contaminates [93]. In rhizofiltration, contaminant clings or assimilates to the roots, and can be transported to the plants. This method is mostly used to sterilize underground wastes or polluted water. Mostly radioactive substances or metals are removed by this method. Abhilash et al. [94] used the phytofiltration technique to increase the Cd uptake from water by using *Limnicharis flava* as an experimental plant. Islam et al. [95] reported the phytofiltration capability of *Micranthemum umbrosum* to remove Cd and arsenic (As) from a hydroponic system. In another experiment, the rhizofiltration potential of *Arunda donax* for Zn and Cd removal, it and recommended the use of the rhizofilteration technique for Cd elimination [96].

It is a cost-effective technique, and plants act as solar-driven pumps to extract the contaminants from the environment [93,95]. However, any contaminant below the rooting depth is not extracted. It is a time-consuming technique and will not suffice for the extraction of both organic and metal contaminants [95,97].

### 3.4. Phtytostimulation

Phytostimulation is a technique used to boost the process of phytoremediation by stimulating the root-released compounds to enhance microbial activities. These exudates enhance microbial growth by fulfilling their nutrient requirements. This process is being used in rhizoremediation technologies. It is a low-cost technique for Cd removal and other organic compounds [98]. Another method is the addition of resistant microbial inoculants into the soil, which can cause the accumulation of heavy metals, including Cd [99].

It is a more effective technique for converting toxic contaminants into non-toxic chemicals. Both in situ and ex situ practices can be done with low-cost treatments [100]. Microbes are able to help limit the growth of plant pathogens and increase nitrogen (N) fixation [101]. However, it is a more time-consuming technique, and the use of volatile and biodegradable compounds ex situ is not an easy practice. The process is sensitive to the level of toxicity in soil, and in some cases, incomplete breakdown of the organic compounds is observed. Moreover, well-controlled monitoring is required for this technique [100].

## 4. Effect of Phytoremediation on Cd Removal from Soils

The phytoremediation of soil contaminated with Cd has been a serious issue worldwide. In phytoremediation, hyperaccumulator plants are of particular importance as they are mainly involved in the uptake of Cd from soil [111]. Different hyperaccumulators vary in their capacity for Cd extraction from soil. This is because of the low affinity of Cd and its mobile nature [111]. The mobility of Cd in soil makes it easily available for the plant to extract it from the soil, which is later transported from the root to the aerial parts [112]. Some of the factors that facilitate the remediation of Cd process are pH, temperature, and the presence of other heavy metals in the soil [113]. For example, *Chromolaena odorata, Gynura pseudochina, Conyza sumatrensis,* and *Nicotiana tabacum* were tested in field conditions. The field soil was heavily contaminated with Cd; however, all the tested hyperaccumulator plants significantly reduced the Cd concentration in soil [114]. Various mechanisms of Cd phytoremediation are discussed comprehensively below.

## 5. Role of Transporters in Cadmium Accumulation on Hyperaccumulator Plants

### 5.1. Long-Distance Cd Transport

**Figure 1:**
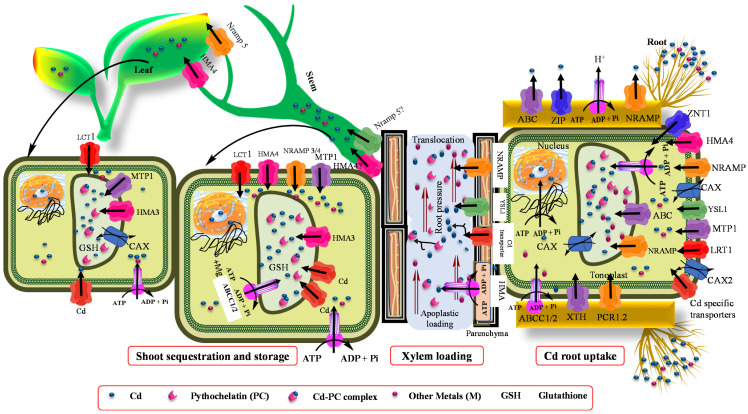
Depiction of major transporters present on the root, shoot, and leaves for Cd sequestration and storage (these processes are related to phytoremediation). Read text and Table 2 for more information.

**Figure 2:**
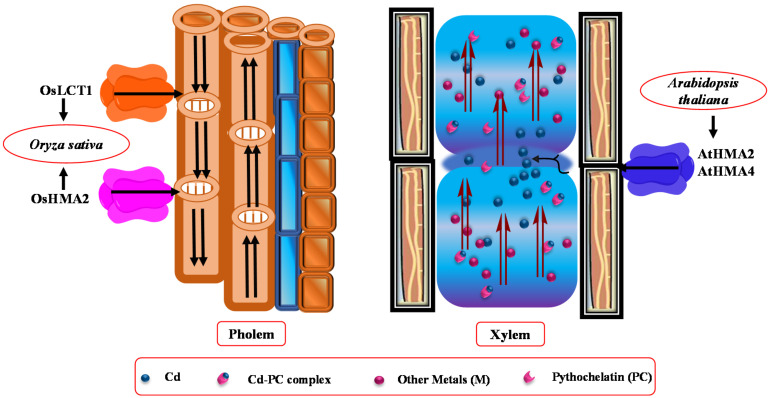
Representation of phloem and xylem transporters. Low-affinity calcium transporter 1 (LCT1) and metal-transporting ATPases (HMA2) that function in the translocation of Cd into the phloem. The major transporters of Cd loading into the xylem are HMA2 and HMA4. However, the role of transporters between the xylem and phloem is unclear.

Accumulation of Cd is regulated by several processes, including vacuolar sequestration, xylem loading, cytoplasm across the membrane, energy-driven transport, cell wall adsorption, and Cd apoplastic influx into root tissues (Figure 1 and Figure 2) [115,116]. One of the proposed prerequisites for bioremediation is that heavy metals are transported to and sequestered in aerial parts. Long-distance transport contributes substantially to maintain a low Cd concentration in roots, and takes part in overflow protection machinery [117,118]. These processes are mediated by several families related to metal and metalloid transport, such as P1B-ATPase [(enzymes that catalyze the hydrolysis of a phosphate (P) bond in adenosine triphosphate (ATP) to form adenosine diphosphate (ADP)] [118,119]. When present in ionic form, Cd transport from root to other tissues is mediated by three major transport system [120,121,122], such as low-affinity calcium (Ca) transporter 1 (TaLCT1) ZIP [(Zn transporter proteins (ZRT)- and Fe-regulated transporter (IRT)-like protein)] transporters, TcZNT1/TcZIP4 and Zn/Fe-regulated transporter-like protein (AtRT), and natural resistance-associated macrophage protein (NRAMP) [123], which includes OsNARMP1, 5, and 6. Moreover, the transport system of Fe uptake is also involved in Cd uptake. Takahashi et al. [123] and Milner et al. [124] also observed that *OsNramp1* enhanced Cd accumulation in the shoot. Furthermore, yellow strip-like 1 (YS1/YSL1) Fe transporters transport Fe in its chelating form. Murata et al. [125] also identified Fe phytosiderophore transporters (HvYS1) in barley, which showed strict specificity for both metals and ligands. In addition, Sasaki et al. [122] and Ishimaru et al. [126] deduced that *OsNRAMP* that plays a key role in Mn^2+^ transport and also showed a major route for Cd transport in rice. From all the above-reported transporters, NRAMP may be involved in several functions, such as metal detoxification, uptake, intracellular transport, and translocation, in many plants [126,127,128,129]. Moreover, the Ca^2+^ blocker also inhibits Cd transport in *Suada salsa*, suggesting their contribution to Cd transport [130]. Collectively, Cd is transported through Zn, Fe, and Ca transporters in plants that include LCT1 and ZIP family (Zn/Fe transporters), especially ZIP-IRT [124], and macrophage protein Nramp channels [42]. Therefore, Table 2 shows the summaries of Cd transporters, their function, and location in plants.

AtPDR8 is an ABC transporter that is involved in metal homeostasis and Cd tolerance, and is mainly localized on the epidermis and membrane of root hairs [131]. Once taken by the roots, Cd is then transported into the xylem and other shoot parts. The transport differences in Cd transport in the xylem and shoot are due to genetic variations [132]. Metal tolerance proteins (MTPs) and cation diffusion facilitators (CDFs) can be involved in this whole process [133,134]. Moreover, the P_18_-type metal transporter ATPase (HMAs) also takes part in Cd transport across the membrane, which is required for metal homeostasis. While working on an *Arabidopsis hma2/hma4* mutant, it was observed that OsHMA (pericycle transporter) also transports Cd [135]. OsHMA2 has been described as a key transporter of vascular Cd [136]. The knockout mutant of OsHMA2 resulted in the reduction of Cd in the shoot and grain, which has been confirmed in several studies [118,119,137,138].

Furthermore, low-affinity cation transporters OsLCT1 load Cd metal into the phloem sap [120]. Glutathione (GSH) and its derivatives, phytochelatins (PCs), showed strong bounding with As, Hg, and Cd (Figure 1) [139]. A Cd complex with PCs was also seen in rapeseed phloem sap. According to Mendoza-Cozatl et al. [139] and Kato et al. [140], GSH-Cd (reduced GSH-Cd complex) contributes to long-distance transport of Cd in the phloem. However, PCs appeared in phloem sap after the application of Cd, and they showed a strong affinity for Zn [141]. Mendoza-Cozatl et al. [139] observed thiol-conjugates in phloem that were transported in different sinks after uptake from ATP-binding cassette subfamily C proteins (ABCC1 and ABCC2). PCs-Cd conjugates were involved in root vacuole Cd sequestration, while the GSH-Cd complex was only detected in the seed source. Moreover, the LCT1 transporters also mediates phloem-based Cd distribution (Figure 2) [120,142].

### 5.2. Vacuolar Storage and Sequestration

Several families of transporters, such as ABCCs, NRAMPs, Ca^2+^ exchanger (CAXs), and HMAs, have been investigated in vacuolar sequestration of Cd [143,144]. Among these, ABCC transport PCs (PCs-Cd conjugated). Similarly, Park et al. [143] reported that ABCC1 and ABCC2 are important vacuolar transporters that confer tolerance to Cd, mainly AtAbCC3 plays a role in PC-mediated Cd tolerance [145]. The NRAMPs transport various divalent metals, such as Zn, Mn, Fe, and Cd. NRAMP3 and NRAMP4 are located on the tonoplast and play an important role in the remobilization of essential metals from the vacuole to the cytosol [146]. The CAXs are tonoplast-localized transporters that have specific transportability of Ca^2+^. However, Korenkov et al. [147] reported that AtCAX2 and AtCAX4 transporters are not only specific to Ca^2+^ but also transport other metals, including Cd. In this consistency, the Cd hyperaccumulator *Arabidopsis halleri* showed Cd tolerance with higher expression of *AhCAXI* [3]. In low-Cd-accumulating rice cultivars, OsHMA3 is functional and able to sequester Cd into vacuoles; however, in high-Cd-accumulating cultivars, it is present in an inactive form due to a single amino acid mutation (Table 2) [148,149]. Moreover, higher *NcHMA3* expression also plays a role in Cd hyperaccumulation in *Noccaea caerulescens* [150]. Furthermore, the Cd hyperaccumulator *Sedum alfredii* also showed a higher expression of SaHMA3 [151]. Currently, Liu et al. [152] has discovered that SpHMA3 is critical for Cd detoxification and vacuolar sequestration in young leaf cells of the *Sedum plumbizincicola* plant. They found elevated expression of SpHMA3 in shoots, while Ueno et al. [150] observed the same expression level of HMA3 in both the shoot and roots. The CDF transporter family is also involved in vacuolar sequestration and storage and transport of metal ions [153].

### 5.3. Mechanism of Cd Crossing the Plasma Membrane of Root

At the root plasma membrane, H_2_CO_3_ dissociates into HCO_3_ and H^+^ trough root respiration, so absorbed H^+^ rapidly exchanges with Cd^+^, and then Cd absorbed on the surface of the root epidermis cells, and its exchange into root epidermis cells layers occurs trough the apoplastic pathway [154]. Roots hairs provide a large surface area for Cd absorption from the soil through diffusion [155]. Plant roots also secrete certain organic compounds, such as chelates, that complex with Cd ions to form ligands, allowing its entry into the root epidermis [156]. Moreover, Cd is also taken up by non-selective cation channels, Zn/Fe-regulated transporters [157], and MTPI [158]. Additionally, certain protein transports, such as NRAMPs [159], P-type ATPase (AtHMA4 and AtHMA9) [160,161], ABC transporters (OsPDR9 and AtPDR8 [131,162], and the CAX family (AtCAX2 and AtCAX5) [163,164], impart a critical role in Cd transport across the root plasma membrane. In general, after Cd uptake by plant roots, the maximum portion of Cd gets fractionated into the roots, and only a small portion gets fractionalized to the upper areal parts [160].

## 6. Antioxidant Defense: A Key Mechanism of Cadmium Tolerance and Phytoremediation

Cadmium inhibits the activity of various metabolic cycles and a non-redox active metal that induces many ROS, including H_2_O_2_, superoxide radicals (O_2_^•−^), and hydroxyl radical (OH^•^). Plants have an established mechanism to eradicate oxidative impairment through the protective antioxidant defense system that includes enzymatic antioxidants (SOD, CAT, APX, glutathione reductase (GR), glutathione peroxidase (GPX), glutathione *S*-transferase (GST)) and non-enzymatic antioxidants (GSH, carotenoids, ascorbic acids (AsA), and tocopherol) [179,180]. Antioxidants’ response against Cd toxicity varies amongst different plant species and experimental conditions [180]. The modulation of antioxidant machinery during the phytoremediation process is important because it prevents the plant from Cd toxicity. For example, some hyperaccumulators have the tendency to uptake/remove an abundance of Cd from the soil, but their physiological and biochemical processes still function properly. This could be because of the high antioxidant enzyme activities, which keeps these hyperaccumulators alive even in the most unfriendly environment. Table 3 documents the experiments showing the potential of the antioxidant defense system as a key mechanism of Cd tolerance and phytoremediation.

In wheat, Cd tolerance is linked with high activity of antioxidant enzymes, photosynthetic rate, and hormone concentrations [181]. Cd stress increases the activity of SOD, CAT, POD, and MDA, and reduces the photosynthetic rate, transpiration rate, stomatal conductance, and auxin, gibberellin, and zeatin nucleoside concentrations in wheat leaves. Shah and Nahakpam [182] found six SOD isozymes in a Cd-tolerant rice cultivar (Bh-1) while only three were found in sensitive (DR-92) rice cultivars, suggesting the SOD improves the tolerance capability of rice against Cd stress. Different plant nutrients, such as silicon, sulfur, and iron, reduce the Cd toxicity in higher plants by affecting its accumulation. Glutamate (Glu; 3 mM) is also involved in the abiotic stress response and has been found to significantly elevate Cd accumulation in rice by up to 44% (root) and 66% (shoot) [169,183]. *Macleaya cordata* has shown a high Cd phytoremediation ability (393 μg plant^−1^) with increased biomass. The high Cd concentration, showing high SOD and MDA activity required, and increased capacity of ROS, proves *M. cordata*’s efficiency in Cd phytoremediation [184]. Phosphorus with 100 µM Cd enhances the activity of SOD, POT, CAT, AsA, and α-tocopherol in wheat and decreases the Cd accumulation in shoots [185]. Sunflower seeds under Cd stress showed reduced biomass, carotenoid, and low chlorophyll concentration with an increase Cd in the shoot and root. Pan et al. [186] examined the genetic insight of high Cd-tolerant *Kandelia obovata*, a mangrove plant, and reported two genes *KoFSD2* and *KoCSD3* that showed greater SOD levels and differentially maintained the reactive oxygen mechanism when overexpressed in *Nicotiana benthamiana* under Cd toxicity.

An increased level of H_2_O_2_ decreases the efficiency of the plant for Cd tolerance; therefore, its accumulation is avoided properly by the action of the oxidoreductase enzymes CAT and POD [187]. An increased content of CAT in the leaf of Cd-tolerant wheat lines reduced the translocation of Cd from the root to shoot whereas the 50 µM Cd enhanced the MDA and reduced the activity of CAT and SOD in leaf [188]; while in *Glycine max*, greater Cd accumulation was observed in root with an increased level of GR (up to 370%) followed by CAT (271%) and SOD (193%) in a tolerant cultivar [189].

Gratao et al. [190] applied the grafted technique in Micro-Tom to analyze the antioxidant response to Cd stress. The grafted plant showed a better signaling response to Cd stress from the root to shoot while the non-grafted plants showed decreased levels of CAT, APX, and GR. In rapeseed, high Cd stress decreased the activity of antioxidant enzymes, i.e., SOD, GR, APX, and CAT, while the lipid peroxidation level increased. Likewise, *Brassica juncea* exposed to Cd stress showed no change in antioxidant activity, with an increased level of NP-SH and PCs, that worked under metal stress [191], or increased GR activity up to 50 µM L^−1^ with more accumulated Cd in the leaf of the sensitive cultivar [192]. Enzyme activity increased with an increase in the Cd concentration (100 mg kg^−1^), such as SOD (81%), CAT (36%), and POX (57%), in *Brassica juncea* as compared to the control [66].

## 7. Chelate-Assisted Cadmium Phytoremediation

Due to the limitations in the phytoextraction technique, the use of chelating agents is considered as a suitable alternative to other conventional methods to remediate contaminated soils [202]. In general, chelates are known as stimulating chelating agents in the release of divalent and trivalent cationic metals into soil water to enhance their absorption by the roots of plants. They are classified into three groups of (i) synthetic amino-polycarboxylic acids (APCAs), such as EDTA, ethylene-glycol-tetraacetic acid (EGTA) and sodium-dodecyl-sulfate (SDS); (ii) natural amino-polycarboxylic acids, including S,S-ethylene-diamine-disuccinic acid (EDDS) and nitrile-triacetic acid (NTA); and (iii) low molecular weight organic acids (LMWOAs) containing oxalic acid (OA), citric acid (CA), and tartaric acid (TA) [203,204,205].

**Figure 3:**
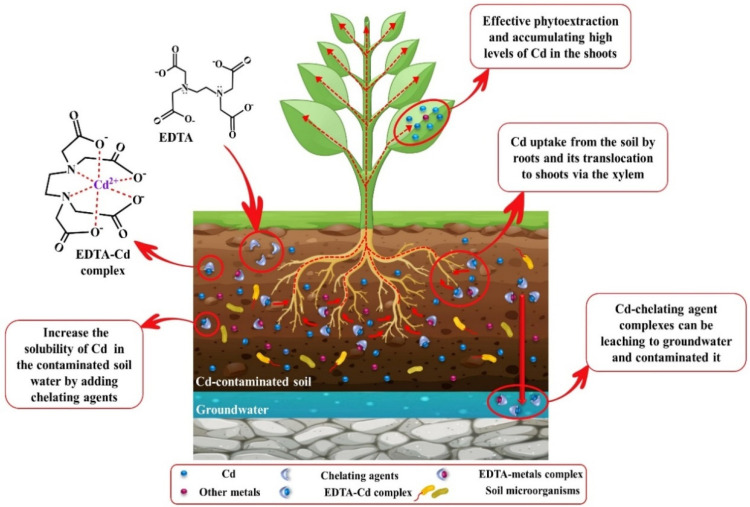
A hypothetical model of phytoremediation by adding chelating agents to remediate Cd-contaminated soil.

Several investigations have been carried out on EDTA as a heavy metals chelator from polluted water and soils for effective phytoextraction [204,206,207]. In several studies, it has been reported that the application of EDTA in contaminated soils leads to elevated Cd accumulation in the aerial parts of plants [207,208]. As stable EDTA-metal complexes have a long-term persistence in soil water, concerns have been raised about the leaching of soil-cationic metal ions and contamination of groundwater and adverse impacts on rhizosphere microorganisms. Therefore, natural chelating agents, such as EDDS, were proposed as a biodegradable chelating agent, a substitute for EDTA [202]. Use of EDDS can decrease metal leaching compared to EDTA but not wholly prevent it [203]. For example, Evangelou et al. [209] described that the half-life of EDDS, depending on the dose of EDDS added to the soil, was between 4.2 and 6.6 days, which is less than EDTA; however, there was Cd and Cu leaching. APCA chelates help to increase the solubility of Cd in soils and efficient phytoextraction but have no role in its elimination. Adding low doses of LMWOAs to Cd contaminated-soil has been suggested as a suitable alternative to other chelants for phytoremediation by (i) the formation of soluble organic acid-Cd complexes, (ii) providing a carbon source for the microorganisms present in the rhizosphere and increasing their diversity; (iii) amending soil quality; (iv) a high degree of biodegradability and low leaching risk; (v) and also improving soil acidity, thus elevating the solubility of Cd ions in soil water [181,210]. In a study on biodegradable chelators, effective phytoextraction of Cd, As, and Pb by *Pteris vittata* in the presence of 1 mM kg^−1^ EDDS compared to OA and CA was identified. However, adding of 2.5 mM kg^−1^ OA improved the soil quality and diversity of soil microorganisms [211]. Moreover, the results of the experiments proved that usually LOWMs in low doses had the highest efficiency of phytoextraction, while higher doses of these chelates negatively affect the Cd uptake and plant biomass. For instance, a study using sunflower plants in Cd-polluted soil showed that Cd absorption at the plant’s roots in the existence of higher levels of CA was much lower than in plants exposed to lower doses of CA [212]. A hypothetical model of phytoremediation by adding chelating agents to remediate Cd-contaminated soil is illustrated in Figure 3.

Generally, the disadvantages of the application of chelating agents in soils can reduce the growth and biomass of the plant, as well as the adverse effects on the soil quality [213]. Moreover, some studies have reported that EDTA elevates Cd mobility in soils and its absorption by the roots, though EDTA is not able to overcome the restrictions of the translocation of Cd from the root to shoot [214,215]. Since the absorbed EDTA-Cd complex moves through the apoplastic pathway, the existence of the *Casparian strip* and suberin deposits disrupt the transfer of this complex from roots to aerial parts, so greater amounts of Cd are accumulated in roots than aerial parts [216,217]. Despite the enhanced levels of soluble Cd in the soil by chelators, regarding the existence of high amounts of Ca and Fe in the soil and their competition for binding to these chelators, higher amounts of these chelating agents must enter the soil to bind to Cd. On the other hand, plants able to absorb only a small portion of the soluble Cd, and a high amount of the soluble Cd-chelate complexes persist in the soil [217]. Notably, the Cd-chelate complex is stable across an extensive pH range, its leaching is unavoidable, and it could enter groundwater and contaminate it [218].

## 8. Phytochelatins and Metallothionein for Cadmium Phytoremediation

**Figure 4:**
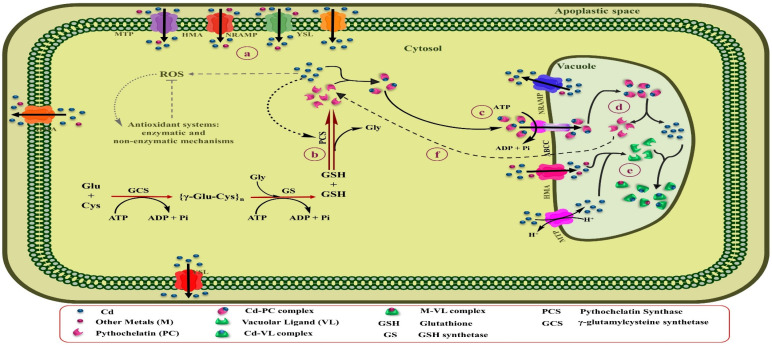
Schematic overview of Cd tolerance and its accumulation by PCs in leaves cells of Cd-hyperaccumulating plants. (**a**) Elevated Cd concentration inside the cytosol. (**b**) The Cd can be directly bound to the PCS and stimulate the biosynthesis of PCs from GSH by activating this enzyme. (**c**) Once the Cd-PCs complexes are formed, they can finally be sequestered within vacuoles by transferring ABC transporters through the tonoplast. (**d**) Due to the low pH of vacuoles, Cd-PCs complexes disassociate, and Cd can be stabilized in vacuoles by binding to ligands, including organic acids and probably amino acids. (**e**) PCs may be by destroyed hydrolase enzymes inside the vacuole or returned to the cytosol, where they able to keep their role as Cd shuttles.

The binding of Cd to high-affinity chelators, including phytochelatins (PCs) and metallothioneins (MTs), and organic acids and amino acids within plant cells is one of the vital adaptative mechanisms [46]. Moreover, the character of these ligands varies, depending on their position inside the plants and plant age [219]. Notably, among metals, Cd is known as a potent stimulator of PCs in different types of plants [220]. Figure 4 represents a schematic summary of Cd tolerance and its accumulation by PCs in leaf cells of Cd-hyperaccumulating plants.

PCs are Cys-rich polypeptides that include duplicate units of γ-glutamyl-cysteine, followed by one glycine (Gly) in the C-terminal [(γ-Glu-Cys)n-Gly] with 2 to 11 repeating units. Albeit, depending on the plant species; Gly in the C-terminal can be substituted by Ala, Ser, Gln, or Glu. These metal ligands are enzymatically synthesized by γ-Glu-Cys dipeptide transpeptidase (PC synthase) from GSH in the cytosol to transfer Cd-PCs complexes to vacuoles or the apoplastic space by ATP-dependent pumps [221,222]. PC synthase is activated by direct Cd binding to the enzyme [223]. While the Cd concentration in the cytosol is elevated, PC synthase enhances tolerance and detoxification of Cd by biosynthesis of PCs [222]. Lee and Hwang [224] found that the overexpression of the *NtPCS1* gene involved in PC synthase biosynthesis in transgenic tobacco plants results in high tolerance to Cd and As, and improved growth and development in these plants. By binding Cd to PCs, it forms a constant complex that is less toxic than the free Cd ions existing in the cells. Once the Cd-PCs complexes are formed inside the cytosol, they can finally be sequestered within vacuoles by transferring the ATP-binding cassette (ABC) transporters through the tonoplast (Figure 4) [225]. So far, three types of vacuolar ABC transporter, AtABCC1, AtABCC2 [143], and AtABCC3 [145], have been recognized in the *Arabidopsis thaliana* that are involved in the transfer of Cd-PCs complexes across the tonoplast into the vacuole and Cd tolerance [226]. Current research has determined that metals compartmentalize as a critical resistance mechanism to reduce oxidative stress and damage to the photosynthetic apparatus in the shoot cells of hyperaccumulator plants [227]. Similarly, it has been identified that mature leaves contain higher amounts of S-rich chelators (PCs and MTs) than in young leaves and can store high concentrations of Cd [228]. Sun et al. [229] found that the levels of PCs in leaf mesophilic tissues of the Cd hyperaccumulator *Solanum nigrum* were dramatically higher than the non-accumulator *Solanum melongena*, which caused the accumulation of significant amounts of Cd and their detoxification in shoots of the Cd-hyperaccumulator population. Similarly, analysis of the EDX spectrum in potato exposed to different Cd concentrations indicated that the highest amount of Cd was stored in the mesophilic tissue of leaves by binding to S-rich compounds [230]. A study on alfalfa under Cd stress conditions indicated that Cd tolerance in seedling roots was enhanced by increasing levels of the expression of genes associated with sulfur metabolism, especially genes implicated in GSH and PCs biosynthesis, also ABC transporters [231]. Due to the low pH of vacuoles, Cd-PCs complexes disassociate, and Cd can be stabilized in vacuoles by binding to ligands, including organic acids and probably amino acids [232]. PCs may be by destroyed hydrolase enzymes inside the vacuole or returned to the cytosol, where they able to keep their role as Cd shuttles [233]. PCs are also implicated in the long-distance transfer of excess Cd from the root to aerial parts to reduce Cd accumulation in roots [234]. For instance, PCs and GSH could play a role as long-distance Cd carriers by the xylem and phloem in rapeseed. Likewise, the rate of Cd transfer by the xylem depends on the Cd concentration in the root [235].

**Figure 5:**
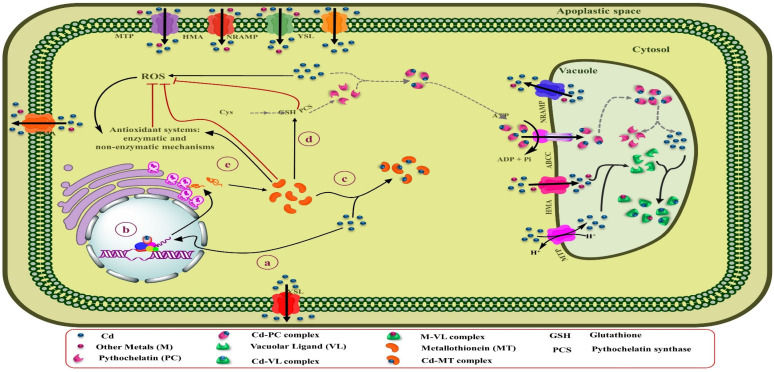
Schematic overview of Cd detoxification and its accumulation by MTs in leaves cells of Cd hyperaccumulator plants. (**a**) By increasing the Cd concentration in the cytosol, (**b**) Cd can stimulate the expression of the MTs gene. (**c**) By binding MTs to Cd, they can play a substantial role in detoxifying and homeostasis Cd within the cytosol. (**d**) Additionally, MTs stimulate GSH biosynthesis as the main constituent of PCs. (**e**) MTs play an essential role in ROS scavenging and its tolerance.

Like PCs, MTs belong to a small Cys-rich protein family with binding sites to metal that exists in an extensive range of organisms [236,237]. In contrast to PCs, which are biosynthesized enzymatically by PC synthase, MTs are directly produced by translating mRNA [238]. MT gene regulation is different in various plants and abiotic stress conditions, including heavy metal-induced expression (Figure 5) [239]. So far, four types of MTs have been recognized in plants, containing MT1, MT2, MT3, and MT4 types, which are categorized according to the arrangement of Cys residues in their C- and N-terminal domains [240]. Regarding the diversity of plants as well as the organization or distribution of Cys residues in the MT structure, there may be different isoforms for each type of MT, e.g., in *A. thaliana*, two isoforms (MT4a and MT4b) have been identified for MT4 [241]. The expression genes of MTs are varied in diverse plant tissues and different growth and development steps of plants [237]. Thus, the genes involved in MT-type1 produce are mostly expressed in roots. In contrast, the genes of MT-type2 are often expressed in the plant shoots and help to store high concentrations of heavy metals in shoots by biding to them, and besides, play an essential role in ROS tolerance. Besides, genes related to MT-type3 are usually involved in fruit ripening and are also expressed in leaves.

In contrast, the expression of genes associated with MT-type4 has been observed in the seed development stage [237]. However, the role of different types of MTs as metal chelators has been identified, which can lead to the tolerance and accumulation of heavy metals in plants [238]. MTs have a great tendency to bind to heavy metals containing Cd, Zn, Cu, and As, and can eliminate them even at a low amount; nevertheless, in terms of the importance as a Cd chelator, MT is considered after PCs [242]. Generally, MTs play multiple roles in plants, such as tolerance and maintenance of the cellular ion balance by detoxification of heavy metals, ROS scavenging, damaged DNA repair, and as well as various physiological processes, including seed germination and fruit ripening in plants [240,243]. However, the mechanism of action of MT plants in stressful conditions relative to mammalian MT remains unknown. It has been established that MT2, in *Coptis japonica*, has 14 Cys-residues in its C- and N-terminal that can bind to four Cd(II) ions [239]. Li et al. [242], proved that in *Ziziphus jujuba* 24 h after Cd exposure, ZjMTs levels elevated and caused increased Cd tolerance and detoxification. This MTs also has six Cys residues in the C- and N-terminal structure and belongs to the MT-Type1 group [242]. It was found that the expression of a gene of *CsMTL2* screened from cucumber fruit was regulated by induction of metal stress, especially Cd in the transformed cells of *Escherichia. coli*. Besides, the highest levels of Cd and Zn were observed consequent of heterologous overexpression of *CsMTL2* in *E. coli* cells compared to the control [242]. In a similar experiment, the same results were obtained on the expression of *CsMT4* screened from cucumber fruit in *E. coli* cells under Cd and Zn stress [244]. Overexpression of *PjMT1* and *PjMT2* genes transferred to tobacco plants from *Prosopis juliflora* caused significantly increased accumulation of Cd in transgenic tobacco plants compared to wild-type plants [245]. Overall, these outcomes suggest that different types of MTs are implicated in the tolerance and accumulation of high amounts of Cd in plant cells. Gonzalez-Mendoza et al. [246] examined Cd and Cu stress in *Avicennia* germinate plants, and they observed a direct relationship between the overexpression of *AvPCS* and *AvMT2* genes and homeostasis and detoxification of Cd and Cu in these plant cells. Additionally, it was shown that the expression of MT genes in yeast strains screened form *A. thaliana*, which were exposed to several metal stress conditions, MT3 was the best candidate for metal phytoremediation [247]. Today, genetic engineering techniques can improve the effectiveness of phytoremediation in plants [248]. For instance, given the importance of vacuoles as a subcellular organelle for storing Cd, the engineering of vacuolar carriers in particular cells, as well as overexpression of proteins in the cell wall with a high affinity to bind to Cd as another site of Cd storage, can help accumulate more Cd in shoots [249]. Further research for understanding the mechanisms of tolerance and accumulation of Cd by genetic engineering techniques might bring a new milestone for the evolution of phytoremediation of Cd by plants.

## 9. Omics Approaches for Cadmium Phytoremediation

Omics technologies, such as genomics, transcriptomics, proteomics, and metabolomics, have been widely applied to study the genetic insights, metabolic pathways, and molecular changes in response to external heavy metal stress response, its transport, and accumulation (Table 4, Table 5 and Table 6).

### 9.1. Genomics

Genomics approaches’ advancement enhances the identification of multiple genes involved in phytoremediation, plant stress tolerance, and transport of heavy metals, such as DNA mismatch repair (MMR) in a soybean cultivar [250], targeted induced local lesions in genomes (TILLING) in rapeseed [8], clustered regularly interspaced short palindromic repeats (CRISPER/Cas9) in rice [251], and genome-wide association studies (GWAS) in rapeseed [252,253].

Navarro-Leon et al. [8] studied the role of the HMA4 gene of *Brassica rapa* through TILLING under 100 µM CdCl_2_ stress. The mutated plant showed increased Cd accumulation in the leaf with an increased level of GSH/GSSG and photosynthetic pigments and reduced biomass and oxidative stress. GWAS using the 60K *Brassica* Infinium^®^ SNP array was performed with phenotypic and genotypic data to understand the mechanism of Cd tolerance in rapeseed. In total, 12 Cd-tolerant genotypes, 9 single nucleotide polymorphisms (SNPs) loci, and 7 genes linked to Cd tolerance were identified [252]. Chen et al. [253] performed GWAS of 419 rapeseed and identified 25 QTLs integrated with 98 SNPs that reside on 15 chromosomes linked to Cd-accumulated traits.

Ma et al. [254] studied *Oryza nivara* to detect quantitative trait loci (QTL) related to Cd tolerance. Seven QTLs residing on chromosome 2, 4, 6, and 8 were identified along with five genes related to oxidoreductase, terpene synthase, carboxypeptidase, and cysteine-rich receptor protein through GeneChip data. Ra44 was obtained as a Cd-tolerant line and further used to study the Cd tolerance in rice. GWAS analysis of 349 wild *A. thaliana* was performed to check the variability in Cd accumulation and the HMA3 locus was found as being potentially responsible for Cd accumulation in the leaf [255].

CRISPER/Cas9 technology was used to obtain three mutants (LCH1, LCH2, and LCH3) of the *OSNramp5* gene from rice that were involved in the uptake of Cd and other metal ions from root cells [251]. Tang et al. [256] used the CRISPER/Cas9 system in the rice indica gene *OsNramp5* to reduce Cd accumulation in rice for food safety, and demonstrated a 0.05 mg kg^−1^ decrease in the Cd concentration whereas the plant yield was not affected.

### 9.2. Transcriptomics

Different technologies are used for transcriptomic studies, i.e., microarray, RNA sequencing (RNA-Seq), cap analysis of gene expression (CAGE), next-generation sequencing (NGS), massive parallel signature sequencing (MPSS), and serial analysis of gene expression (SAGE), as shown in Table 4.

Wang et al. [257] studied the adaptability of the ornamental plant *Verbena. bonariensis* under Cd stress. Plants transcriptome analysis under Cd stress revealed 237,866 transcripts and 191,370 unigenes whose enrichment analysis revealed differentially expressed genes (DEGs) from all major process, especially lignin synthesis, anthocyanins synthase (ANS), and chalcone synthase (CHS), under Cd stress, confirming the plant has a great Cd phytoremediation property through Cd tolerance and distillation of it. *B. juncea* is a well-known plant used for phytoremediation studies utilized for microarray analysis to understand the functional genes associated with Pb and Cd stress. The microarray chip of *A. thaliana* probes was used to study the DEGs in roots of *B. juncea* (variety P78) [258].

The genome-wide transcriptomic profile of *Brassica rapa* var. parachinesis (Chinese flowering cabbage) was performed using Solexa sequencing. They identified 1404 upregulated genes and 1669 downregulated genes, and precisely three Cd tolerance genes identified as HMA3, HMA4, and Nramp1 [259]. In a recent study, Cd accumulation and distribution was investigated in the peanut plant through RNA-seq analysis. Here, 8cDNA libraries identified 4484 novel genes from which 6798 were grouped to Cd-related DEGs among two cultivars, first Fenghua 1 (low-Cd) and second Silihong (high-Cd). A total of 183 DEGs were to be found linked to ion transport-related proteins (ZIPs, MTPs, Nramps, and YSLs), among them 9 genes related to the cell wall and 9 genes specifically related to metal transport from which 4 were linked to endomembrane-tonoplast transported genes (MTP4, ABCC4, ABCC15, and ZIP11), four Cd efflux genes (YSL7, FRD3, PDR12, and ZIP1), and one Cd influx gene (IRT1); higher expression in Fenghua 1 might be related to the difference in Cd transport and accumulation among the two cultivars [260]. Creeping bentgrass transcriptional analysis showed four transcription factors (bZIP, WRKY, MYB, and ERF) linked with cd stress [261].

RNA-seq integrated with PacBio ISO-seq was developed to study the full transcriptomic data of Italian rye grass, a potential phytoremediation plant. Out of 2367 DEGs, the overexpression of the *LmAUX1* gene significantly enhanced the Cd concentration in *A. thaliana*. The transcriptome analysis helped in the construction of full-length UniTransModels; out of this, 26.76% had isoforms [262]. RNA-seq analysis of Pokeweed revealed 10.63 Gb transcriptomic data consist of up to 97,000 unigenes covering 72 metabolic pathways. It had excellent phytoremediation ability, and different heavy metal-tolerance genes were identified, including nicotianamine synthase (8), ABC transporter (3), expansins (11), metallothioneine (3), ZRT/IRT protein (4), and aquaporins (4) [263].

### 9.3. Proteomics

Novel proteomic techniques providing a better understanding of the Cd-responsive proteins for plant stress improvement and phytoremediation are two-dimensional electrophoresis (2DE), sodium dodecyl sulfonate-polyacrylamide gel electrophoresis (SDS-PAGE), matrix-assisted desorption ionization-time of flight (MALDI-TOF), inductively coupled plasma mass spectroscopy (ICP-MS), liquid chromatography-tandem MS (LC-MS/MS), high-performance liquid chromatography (HPLC), etc., to quantify large-scale proteomic profiles (Table 5).

Proteomic analysis of xylem tissue of Cd-treated rapeseed plant revealed the proteins related to energy production, carbohydrate metabolism, and redox reduction [267]. Cd stress caused a reduction in growth and photosynthesis when *Populus. tremula* x *Populus. alba* was exposed to 360 mg kg^−1^ Cd for 61 days [268]. Proteomic analysis (MALDI-TOF/TOF, 2DE) of a Cd-treated *Sorghum bicolor* plant revealed increased expression of 15 proteins while it downregulated 8 proteins. Mostly, Cd reduces the activity of ATP production, carbon fixation, and protein synthesis regulation [269]. Suspension cells of rice were subjected to iTRAQ and ICP-MS analysis to study improved Cd stress with silicon [270].

Under 500 µM Cd stress, *Microsorum pteropus* fern had a higher ability to hyperaccumulate Cd (up to 4000 mg kg^−1^) in its root and leaf dry mass. Proteomic analysis of fern leaves and roots through MALDI-TOF revealed eight proteins majorly involved in energy metabolism. It enhanced the antioxidant activity to reduce damage from Cd, whereas 20 proteins differentially expressed in leaves were mainly involved in the regulation of photosynthesis and cellular metabolism [271].

### 9.4. Metabolomics

Different analytical approaches have been developed to understand the plant metabolic response, including nuclear magnetic resonance spectroscopy (NMR), gas and liquid chromatography (GC and LC), and inductively joined mass spectrometry (Table 6). Amino acids, phenols, carotenoids, α-tocopherol, and glutathione are major metabolites whose synthesis varies under metal stress. Cd stress caused a vital change on the metabolism of amino acids, sugar, and organic acids [278,279]. Siriporansdulsil et al. [280] reported an increased level of proline under Cd stress in microalgae whereas an increased level of α-tocopherol was observed in *A. thaliana* under Cd stress [281]. Metabolic pathways and metabolite enhance Cd tolerance and absorption by plants by promoting PCs [282].

LC-MS/MS and HPLC analysis for metabolites and thiol compounds in *Amaranthus hypochondriacus* revealed that the plant accumulates 40 times more Cd inside leaves as compared to the control under Cd stress. Among 41 SDMs, 12 were significantly related to PCs in 3 metabolic pathways as Lue, Val, and Ile biosynthesis; Asp, Ala, and Glu metabolism; and Pro and Arg metabolism [282]. Navarro-Reig et al. [283] identified 112 metabolites in rice through LC-MS (HPLC joined with Q-Exactive mass spectrometer). Mengdi et al. [284] identified nine metabolic pathways in *A. hypochondriacus* (K472) through LC-MS analysis. The intermediate vegetative stage had the highest Cd tolerance ability, which expressed 29 significantly different metabolites involved in 4 metabolic processes, including purine metabolism; Gly, Thr, and Ser metabolism; Asp, Ala, and Glu metabolism; and Arg and Pro metabolism. Further, 100 μM Cd caused an increase in asparagine, tyrosine, and α-tocopherol levels in the leaves of a tomato plant but overall causes severe metabolic and physiological deformities in the plant whereas tomato could bear the exposure to 20 μM Cd adequately [285]. In radish, GC-MS analysis revealed that Cd stress caused an alteration in amino acid metabolism, energy production, and oxidative phosphorylation pathways [278].

## 10. Genetic Engineering for Cadmium Phytoremediation

A series of studies addressed the issue of remediating Cd through genetically engineered plants (Table 7) as it is known that metals with similar chemical and physical properties biologically antagonize each other [288]. Interestingly, there is a high resemblance between the ionic hydrated radius of Fe^2+^ (4.28 a.m.) and Cd^2+^ (4.26 a.m.) [248]. In line with this, a *bts-1* lack-of-function mutant in *Arabidopsis* displayed a higher Cd accumulation capacity in its roots and shoots in comparison to wild-type plants. The enhanced Cd accumulation in the roots and shoots of the *bts-1* mutant was because of the positive regulation of Fe nutrition via the upregulation of Fe-related genes [248]. In another study, the detoxification and accumulation of Cd in rice shoots was investigated. The *CAL2* gene, which is located in the cell wall, has shown a good Cd chelation ability. To confirm this, ectopic overexpression of *CAL2* in *Arabidopsis* and rice strongly induced their Cd accumulation capacity without affecting the uptake of other essential nutrients [289]. However, the *CAL2* gene-overexpressing transgenic lines showed sensitivity to Cd stress. The reason could be the high abundance of accumulated Cd in the shoots of transgenic plants [289]. The *Arabidopsis AtPDF2.5* was triggered when the plant was subjected to Cd stress. The results showed the responsive nature of *AtPDF2.5* to Cd [35]. The *AtPDF2.5* possessed eight cysteine residues, which are involved in the tolerance and chelation of Cd. To validate its role, the *AtPDF2.5* gene-overexpressed plants were generated, which displayed tolerance to Cd and also enhanced the Cd accumulation in shoots. Further, *AtPDF2.5* facilitates Cd efflux in the cytoplasm via its chelation activity [35]. Similarly, the *Arabidopsis AtPDF2.6* possessed Cd chelation properties and were also induced significantly under Cd treatment [290]. Taken together, these lines of evidence affirmed the importance of genetic engineering in the production of Cd-tolerant crops, which could also be helpful in the phytoremediation of soil.

## 11. Employing Microbes for Cadmium Phytoremediation

**Figure 6:**
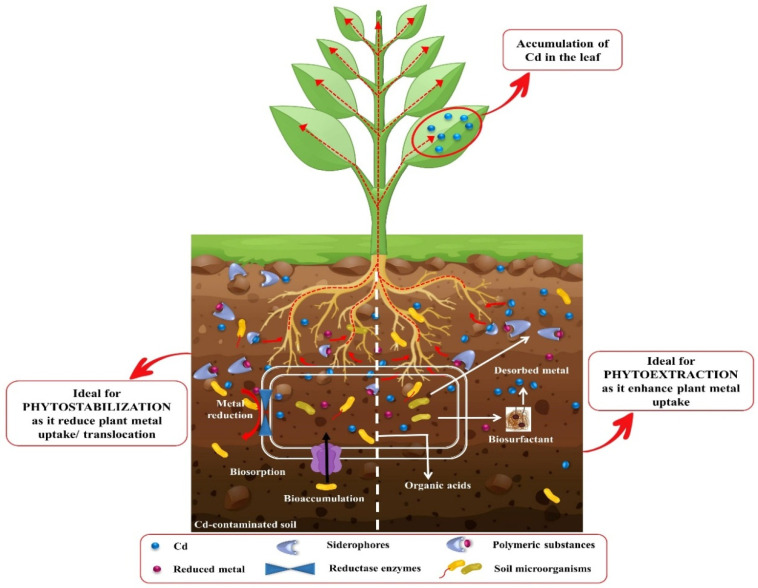
Schematic illustration of Cd phytoremediation by microbes. In the phytoextraction process, the production of siderophores and organic acids by the soil microbes influences the phytoextraction capacity of the accumulator in a positive manner. The generation of polymeric substances by soil microbes keeps the Cd metal in a static form, which facilitates the biosorption and bioaccumulation process.

Transformation of heavy metals from an unavailable to available form is an important factor that decides the fate of phytoremediation. Numerous microbes have been reported to initiate the phytoremediation of Cd by fractionating it in the soil and allow the plant to uptake it. Different classes of microbes (bacteria and fungi) play a crucial role in Cd phytoremediation by activating various mechanisms and producing different compounds, such as siderophores, organic acid, polymeric substances, bioaccumulation, and biosorption (Figure 6). The siderophores are the iron chelators with la ow molecular weight and generally help in the phytoremediation of Cd [297]. Organic acids, which enhance the bioavailability of Cd, are produced by soil microbes. Additionally, organic acid influences the soil pH level by keeping it low, which facilitate the phytoextraction process and is thus crucial for the phytoremediation of Cd [298]. The secretion of extracellular polymeric compounds (exopolysaccharides, mucopolysaccharides, and glomalin) reduces the mobility and bioavailability of Cd, which makes it an essential element in the Cd phytoremediation process [299]. Biosorption and bioaccumulation refer to the processes of metal absorption and accumulation by plants with the help of soil microbes [300]. The biosorption and bioaccumulation contribute largely to Cd phytoremediation by phytostabilizing Cd at the root zone. Below, we discuss the role of bacteria and fungi in promoting phytoremediation of Cd in soil.

### 11.1. Role of Arbuscular Mycorrhizal Fungi

The arbuscular mycorrhizal fungi (AMF) is a portent regulator of plant growth under various stress conditions, including heavy metals. In general, the AMF makes a symbiotic association with the host plant by increasing the availability of solubilized P [301]. According to previous research, the uptake of Cd largely depends on the amount of accumulation of P [301]. This suggests the critical role of phytoremediation of Cd by AMF. For instance, the application of AMF in Cd-polluted soil significantly enhanced Cd accumulation and phytoavailability in the root and shoot tissues by lowering the soil pH and chemical fractions [302]. Additionally, it increased the tolerance of *Solanum nigrum* to Cd stress [302]. *Eichhornia crassipes* is considered as a metal hyperaccumulator and plays an important role in soil phytoremediation. The combination of *E. crassipes* with AMF reduced the Cd concentration in soil to a large extent. Additionally, the inoculation of AMF with E. crassipes substantially induced Cd accumulation and translocation in the roots and shoots [303]. In Table 8, we list studies featuring the role of AMF and other strains of fungi in Cd phytoremediation.

Therefore, this phytoremediation strategy can be applied to minimize the Cd toxicity in soil and water [303]. The growth of Cassia italica Mill has been restricted by the increased amount of Cd in soil [304]. The inoculation of the AMF to *Cassia italica* Mill medicinal plant significantly hastened the tolerance to Cd by limiting its translocation to shoots. Meanwhile, a higher amount of Cd uptake was observed in the roots of the *C. italica* Mill plant inoculated with AMF [304]. Tomato fruits are mainly consumed throughout the world; however, the Cd toxicity severely affected the growth and yield of it. On the other hand, the accumulation of high amounts of Cd in tomato fruit could be extremely harmful to human health [305]. The inoculation of AMF in tomato plants reduced the Cd toxicity and prevented the translocation of Cd from the roots to the aerial parts of the plants [305]. The application of Cd could be a useful strategy to protect hazardous environmental changes, such as the removal of Cd from the soil, and reduced the usage of inorganic fertilizer.

### 11.2. Role of Plant Growth-Promoting Bacteria (PGRB) in the Phytoremediation of Cd

A plethora of studies highlighted the role of PGRB in the phytoremediation of toxic metals, including Cd (Table 9). Among the group of PGRB, some of them detoxify and break down heavy metals by releasing various essential binding compounds, such as organic acid, siderophores, exopolymers, and biosurfactants, which makes the metals available to the plants (Figure 6) [316]. For example, *Arthrobacter* inoculated to *Ocimum gratissimum* helps in the removal of Cd from the soil by inducing the uptake of Cd through roots [317]. Similarly, the *Arthrobacter* sp. was inoculated to the *Glycine max* under Cd-contaminated soil. The application of *Arthrobacter* sp. remarkably enhanced the bioavailability of Cd to the plant. Additionally, increased accumulation of Cd in the roots was also observed in the *Arthrobacter* sp.-inoculated plants [318]. The *Bradyrhizobium* sp. is a Cd-tolerant PGRB that reduces Cd toxicity and improves agronomic traits of plants grown in Cd-contaminated soil [319]. The inoculation of *Bradyrhizobium* sp. over *Lolium multiflorum* triggered the uptake of Cd from the soil and increased the biomass. The induction in Cd accumulation was also observed in the shoots of *Lolium multiflorum* [319]. The *Mesorhizobium huakuii* subsp. rengei B3 was inoculated to an *Astragalus sinicus* plant. The results showed a 19-fold increase in the cell-mediated Cd^+2^ binding capacity [320]. In rapeseed, the *Arthrobacter* sp. SrN1 and *Bacillus altitudinis* SrN9 was applied to alleviate the deleterious effects of Cd. The *Arthrobacter* sp. SrN1 and *Bacillus altitudinis* SrN9 inoculation not only boosted the resistance of rapeseed to Cd stress but also increased the uptake and translocation of Cd [321]. This indicates the potential role of *Arthrobacter* sp. SrN1 and *Bacillus altitudinis* SrN9 in Cd phytoremediation without altering the plant productivity. Likewise, enhanced Cd uptake was observed in the roots of the *Sedum plumbizincicolaa* plant after being inoculated with the bacterial strain *Rhodococcus erythropolis* NSX2 [322]. The application of PGRB to soil could be a useful, cost-effective, and environmentally friendly strategy of Cd phytoremediation. However, more work is required to understand and explore the different strains of microbes involved in the phytoremediation of Cd without hindering plant growth.

## 12. Conclusions and Future Perspectives

Phytoremediation of Cd provides a way forward for the restoration of the polluted environment and has provided many positive and desirable results. Through an extensive literature review, it is evident that Cd interferes with plant functions and as an external stimulus; it activates the defense mechanism through various physiological and metabolic pathways. The multiple phytoremediation strategies offer cost-efficient optimal prospects for the in-situ remediation of Cd in a most environment-friendly way. For a successful rehabilitation, it is essential to utilize the prominent physiological features of Cd hyperaccumulators for the extraction, transformation, and/or stabilization of Cd. Meanwhile, it is important to evaluate the effectiveness of phytoremediation technologies and integrate the available resources. This will not only facilitate the phytoextraction process but also boost the plant productivity in areas with suboptimal soil metal levels by utilizing multi-omics approaches, microbes’ potential, amendments (like AMF and PGPBs), and techniques, such as genetic engineering. Furthermore, the phytoremediation potential and improved tolerance to Cd could be counted as a first step towards leveraging the accumulation potential of plant species. On the other hand, it is equally important to consider the antagonistic and synergistic behavior of contaminants for remediation potential and ensure an ecologically responsible alternative for further use and/or processing of plants, which must be done under a strictly controlled environment.

Notably, the phytoremediation tool is still under the examination and progress phase, and numerous technical barriers need to be resolved. The multifaceted connections that occur under site-specific environments demand a multi-disciplinary approach for metal phytoextraction. This accomplishment will eventually depend upon utilization of a complete analytical tool to integrate the works of plant scientists, soil microbiologists, agronomists, and environmental engineers. Nevertheless, phytoremediation promises to be a vital waste managing choice for the present century.

Moreover, research is required to understand the molecular mechanism of hyperaccumulators in field trials at different locations, as a majority of the current research is based on lab studies. The molecular mechanism of soil amendment-mediated phytoremediation is also unclear. Therefore, omics approaches could be employed to elaborate on how AMF and PGPBs induce the phytoremediation of Cd by regulating numerous genetic, metabolic, and hormonal pathways. An exploration of different genetic pathways will not only enhance the phytoremediation capacity of food crops but also improve their productivity in Cd-contaminated soil.

## Figures and Tables

**Table 1:** Types of phytoremediation approaches and their specific methods. Abbreviations are explained in the text.

Types	Process	Mechanism	Plants	References
Phytoextraction/ Phytoaccumulation	Bioaugmentation-assisted phytoextraction	Combined with mycorrhiza	*Suaeda salsa* and *Trichoderma asperellum*	[102]
	Chelated-assisted phytoextraction	Chelates like EDTA, SDS, and EGTA	*Fagopyrum esculentum*	[103]
	Phytomining	Phytoextraction for commercial use, like silver (Ag), Ni	*Alyssum murale, Odontarrhena chalcidica*	[104]
Phyostabilization	Organic fertilizers, biochar	Immobilization of Cd by using biomolecules	*Virola surinamensis*, *Boehmeria nivea*	[86,91]
Phytofiltration	Biosorption	Metals are absorbed bound in cells, used for phytoremediation	*Lythrum salicaria*	[105]
	Rhizofiltration	Metals are absorbed and bound on only roots	*Micranthemum umbrosum*	[95]
	Blastofiltration	Metals are absorbed and bound on only seedlings	*Moringa Oliefera, Cucumis melo, Abelmoschus esculentus, Ricinus communis*	[106,107]
	Caulofiltration	Metals are bond and absorbed on excised plant	*Berkheya coddii*	[108]
Phytostimulation	Fungi, bacteria	Phytoremediation with the intervention of microorganisms in different terms to remediate soil with organic pollutants	*Rumex K-1* (*Rumex patientia* × *R. timschmicus*) *Viola baoshanesis. Vertiveria zizanioides*	[109,110]

**Table 2:** Summary of Cd transporters; their localization and function in plants.

Transporter	Localization	Function/Substrate	References
Plasma Membrane			
OsNRAMP1	Roots and shoots	Influx of Cd, Al	[123]
AtGLRs	Roots	Cations transporter	[130]
OsNRAMP5	Roots	Uptake of Mn and Cd	[126]
AtCNGCs	Roots	Cations transporter	[130]
OsLCT1	Leaves, nodes, phloem parenchyma	Efflux of Cd, Ca, Mg, and Mn	[142]
CsMTP9	Roots endodermal cells	Efflux of Cd and Mn	[134]
HvNRAMP5	Roots	Uptake of Mn and Cd	[129]
SnYSL3	Vascular and epidermal cells of the roots and stems	Transport of nicotianamine complexes containing Fe(II), Cu, Zn, and Cd	[165]
OsYSL2	Vascular bundles, roots	Influx of Mn and Cd	[166]
AtNRAMP6	Young leaves, roots	Mn and Cd influx	[167]
OsZIP8	Roots	Influx of Zn, Cd	[168]
OsIRT1/2	Roots	Uptake of Cd	[169]
Tonoplast			
AtABCC1/2	Roots and shoots	PCs uptake	[170]
AtNRAMP4	Leaves, roots, vascular bundles	Cd and Fe efflux	[171]
TgMTP1	Roots and leaves	Transporter for Zn and Cd	[172]
AtMTP1	Leaves and roots	Zn and Cd transporters	[173]
OsNRAMP3	Vascular bundles, roots, leaves	Uptake of Mn	[136]
OsHMA5	Roots, vascular bundles	Loading of Cu in xylem	[174]
OsMTP8.1	Roots	Sequestration of Mn into vacuoles	[175]
AhCAX1	Shoots	Uptake of Cd	[3]
AtABCC3	Roots and shoots	PCs uptake	[145]
SpHMA3	Roots, shoots	Sequestration of Cd into vacuoles	[152]
AtCAX2/4	Roots	Cd vacuolar transport	[176]
OsHMA3	Roots	Cd sequestration in root vacuoles	[177]
Others			
AtNRAMP3	Mesophyll vacuoles	Cd, Fe, Mn efflux	[171]
NcHMA3	Root pericycle	Cd vascular sequestration, high substrate specification	[150]
GmHMA3	Roots	Divalent transport	[178]
SaCAX2	Leaves	Cd uptake	[144]

**Table 3:** Summary of experiments showing the potential of the antioxidant defense system as a key mechanism of cadmium tolerance and phytoremediation. Abbreviations are explained in the text.

Plant Specie	Cd Level and Duration	Antioxidant Response to Cd Stress	References
*Lolium perenne*	0.2, 0.5 mM Cd; 7 d	↑POD, CAT, SOD, APX, GPX and GR	[193]
*Brassica juncea*	25, 50, 100, 150 mg kg^−1^ CdCl_2_ soil; 30 days after sowing (DAS)	↑CAT, APX and GR	[194]
*Brassica napus*	0.03, 0.3 and 1 mg CdCl_2_; After three weeks, samples were harvested	↑CAT, APX, GR, GSH and AsA	[195]
*Triticum aestivum*	10, 20 kg ha^−1^ P with 100 µM Cd; 28 d	↑SOD, POD, CAT, AsA and α-tocopherol levels	[185]
*Macleaya cordata*	10–60 mg kg^−1^ Cd soil + 30–180 mg artificially added Cd; 60 d	↑SOD and MDA ↓CAT activity	[184]
*Atriplex halimus*	50, 200, 400, 650 and 1350 µM CdCl_2_; 22 d	↑SOD, CAT, guaiacol peroxidas	[196]
*Paspalum scrobiculatu;* *T. aestivum*	1000 µM CdCl_2_; 14 d	↑SOD and POD	[197]
*Solanum tuberosum*	18 different Cd levels and best selected was 218 µM CdCl_2_; 20 d	Differential activities of CAT, SOD, GPX and APX ↓Lipid peroxidation, H_2_O_2_	[198]
*Glycine max*	50, 100, 150 mg L^−1^; 35 d	↑GR, CAT, SOD	[189]
*Petroselinum hortense*	75, 150, 300 µM CdCl_2_; 15 d	↑POD increased ↓SOD, CAT and APX activities	[44]
*Solanum lycopersicum*	35 μM CdCl_2_; 1, 4 and 7 d	↑APX, GR and GST ↓GSH content	[4]
*Abelmoschus manihot*	5, 15, 30, 60 and 100 mg kg^−1^ CdCl_2_; 60 d	↑SOD, CAT, POD	[52]
*Brassica chinensis*	20 μM Cd; 7 d	↑SOD, CAT, POD, AsA and GSH	[199]
*Quercus robur*	10, 50 µM CdCl_2_; 1 h,3 h, 24 h, 3 and 7 d	↑APX, SOD and CAT activity	[200]
*Sedum alfredii*	100 μM CdSO_4_; 6, 24, 36, 48, 72 h	↑SOD, GR, GSNOR and γ-ECS ↓POD, CAT and APX	[201]
*Oryza sativa*	80 μM Cd_2_SO_4_ with 1, 3 or 6 mM Glu; 1 or 2 weeks	↑CAT, POD and GST	[169]

**Table 4:** Summary of experiments directed using transcriptomic approaches under Cd stress in plants.

Plant Species	Cd Level and Duration	Specific Tissue	Approach	Functional Annotation Methods	Differentially Expressed Genes (DEGs)	Key Findings	References
*Brassica parachinensis*	50 µM CdCl_2_; 3 d	Roots	RNA-seq	GO, KOBAS	3073 DEGs	10 genes selected for qRT-PCR Cd tolerant genes HMA3, HMA4 and Nramp1 identified. Novel pathways viral carcinogenesis and Parkinson’s disease might be potential in Cd tolerance	[259]
*Arachis hypogaea*	2 µM CdCl_2_; 7 d	Roots	RNA-seq	GO, KEGG	2793 DEGs; 331 genes in Fenghua 1; 1302 genes in Silihong	16 DEGs expression validated by qRT-PCR. 9 genes related to metal transport identified (PDR1, IRT1, ZIP1, ZIP11, ABCC4, ABCC15, YSL7, MTP4 and DTX43) in low Cd cultivar might be linked to Cd accumulation and transport	[260]
*Agrostis stolonifera*	2.5 mM (BT2_5); 43mM (BT43) CdCl_2_; 7 d	Leaves	RNA-seq	NR, NT, Pfam, GO, KEGG, KOG	279 DEG (BT2_5); 2509 DEGs (BT43)	9 DEGs selected for qRT-PCR. WRKY, ERF, bZIP and MYB transcription factors linked with Cd stress response	[261]
*Landoltia punctata*	20 µM CdCl_2_; 0, 1, 3, 6, 9 d	Plant	RNA-seq	Pfam, Nr NCBI, COG, Swis-Prot, KEGG, GO	9461 DEGs in CT1; 9847 DEGs in CT3 and 9615 DEGs in CT6.	15 DEGs selected and validated by qRT-PCR. Sulfur and ROS metabolism genes upregulated along with DNA repair, RNA and protein metabolism and carbohydrate metabolic flux	[264]
*Lolium multiflorum Lam.*	50 µmol L^-1^ CdCl_2_; 6 h	Roots	PacBio ISO-seq + RNA-seq	Nr NCBI, Swiss-Prot, GO, KEGG	2367 DEGs	20 DEGs for qRT-PCR construction of UniTransModel. *LmAUX1* gene overexpression enhances Cd phytoremediation in *Arabidopsis*	[262]
*Siegesbeckia orientalis*	50 µM CdCl_2_; 1, 3, 5 d	Roots	RNA-seq	Nr NCBI, COG, pfam, Interpro, Swiss-Prot, GO, KEGG	5896 DEGs C1T1; 3036 C3T3 5149 C5T5	6 genes selected for qRT-PCR. DEGs majorly evolve in Cd absorption and transport, ABC signaling, phenylpropanoid biosynthesis and ROS scavenging pathways	[265]
*Oryza sativa*	15 mg kg^−1^ CdCl_2_; 4 months	Panicle nodes, Node1, Stem node (fresh, leafless stem, snap)	RNA-seq, miRNA	miRNA analysis (Rfam, cDNA seq, miRBase), GO, KEGG	4533 DEGs, 70 miRNA	14mRNA, 3miRNA selected for qRT-PCR. 5 genes *OsIRT1, OsVIT2, OsABCC1, OSNRT1.5A, OSNramps5* and Panicle node and node1 are involved in blocking Cd upward transport	[266]

**Table 5:** Summary of experiments directed using proteomics platforms under Cd stress in plants.

Plant Species	Cd Level and Duration	Specific Tissue	Extraction Protocol	Analytical Approach	Key Findings of Differentially Expressed Proteins (DEPs)	References
*Arabidopsis thaliana*	100 mM L^−1^ CdCl_2_; 30 d	Leaves	Phenol extraction	LC-MS/MS	56 DEPs identified Protein linked to disease resistance, defense, energy and metabolism Increase in activity of APX, CAT and GR, MDA activities	[272]
*Oryza sativa*	10,100 µM CdCl_2_; 12 d	Leaves	Phenol extraction	LC-MS/MS, iTRAQ, qRT-PCR	482 DEPs identified Phosphorylated proteins involved in stress tolerance, transcription factor, signaling and neutralizing reactive oxygen specie	[273]
*Cucumis sativus*	100 µM CdCl_2_; 10 d	Leaves	TCA/Acetone	iTRAQ, LC-ESI-MS/MS	1106 DEPs identified in three contrasting groups (377, 297 and 432). Nitic oxide treatment reversed the Cd disruptive pathways as redox homeostasis, photosynthesis, glutathione mediated Cd detoxification, chlorophyll metabolism, Cd transport and localization and Ca^2+^ signaling transduction	[274]
*Microsorum pteropus*	100, 250, 500 µM CdCl_2_; 7 d	Leaves, Roots	TCA/Acetone	2DE, MALDI-TOF/TOF-MS	8 DEPs identified in roots with enhanced antioxidants and energy metabolism activities 20 DEPs identified in leaves with enhanced photosynthetic functions and regulate cellular metabolism	[271]
*Solanum lycopersicum*	35 µM CdCl_2_; 4 d	Roots, Shoots	Phenol extraction	LC-MS/MS	4051 non-redundant proteins. 358 DEPs in sensitive genotype. 84 DEPs in the tolerant genotype Proteins identified related to peroxidase, GST, cytoskeleton, S-adenosylmethionine (SAM) biosynthesis, glycolosis, chaperone and oxidoreductase.	[275]
*Sphagneticola trilobata*	0.5, 5, 25, 35, 50 µM CdCl_2_; 96 h	Roots, Leaves, Stem	DTPA/TFA soln. for thiol, TFA/acetonitrile for PCs	SDS-PAGE, HPLC	1306 mg kg^−1^ Cd content increases in roots, 193 mg kg^−1^ in stem, 52 mg kg^−1^ in leaves Plant roots has increase content of L-cystein, glutathione, phytochelatin, and thiols	[276]
*O. sativa*	100, 200 mM CdCl_2_; 48 h	Leaves	Phenol extraction	SDS-PAGE, iTRAQ labeling, RP-HPLC, LC-MS/MS	109 DEPs identified in *Bancanggengnuo* (*japonica*), 209 DEPs identified in *Yuzhenxiang* (*indica*) Proteins identified linked to carbohydrate metabolism, redox reactions and signal transduction	[277]

**Table 6:** Summary of experiments directing metabolomics profile under Cd stress in plants.

Species	Stress Condition	Specific Tissue	Analytical Platform	Data Analysis	Key Findings	References
*Solanum lycopersicum*	20, 100 μM CdCl_2_; 90 d	Leaves	NMR analysis, HPLC-PDA	PCA	28 significantly different metabolites (SDMs) metabolites identified Cd stress cause decrease in ascorbate and proline content Cd stress cause increase in α-tocopherol, tyrosine and asparagine accumulation.	[285]
*Raphanus sativus*	40 mg L^−1^ CdCl_2_; 72 h	Tap roots	GC-MS	PCA, PLS-DA, KEGG	1,104 different types of metabolites identified Cd stress cause variations in amino acid metabolism, Energy production and Oxidative phosphorylation	[278]
*Oryza sativa*	10, 50, 100, 1000 μM CdCl_2_; Upto harvest	Aerial parts, Roots	LC-MS	PLS-DA, MCR-ALS, KEGG	Secondary metabolism pathways and amino acid, carbon, glycerolipid and purine pathways affected 112 SDMs detected 97 SDMs was confirmed under Cd toxicity	[283]
*Amaranthus hypochondriacus*	100 mg kg^−1^ CdCl_2_; 20 d	Leaves	LC-MS/MS HPLC	PCA, KEGG, HMDB, MetaboAnalyst	41 SDMs involved in 7 metabolic pathways were detected 12 metabolites identified linked with phytochelatin in 3 pathways, i.e., Val, Leu and Ile biosynthesis, Ala, Asp and Glu metabolism, and Arg and Pro metabolism	[282]
*Amaranthus hypochondriacus*	25, 200 mg kg^−1^ CdCl_2_; 20, 40, 60 d	Leaves	LC-MS	PCA, OPLS-DA, KEGG, HMDB, MetaboAnalyst	SDMs produced as: 31 at early vegetative stage, 29 at the intermediate stage, and 30 at late stages of plant growth Purine metabolism is the primary target of Cd absorption	[284]
*Elodea nuttallii*	280 μg L^−1^ Cd as (Cd(NO_3_)_2_; 24 h	Cytocol, Shoots	GC-MS, LC analysis	PCA, MetaboAnalyst KEGG	Cd stress caused significant variations in aminoacyl-tRNA biosynthesis and branched-chain amino acids pathways In shoot, Cd induce concentration of 11 amino acids, 2 sugars, adonitol and pipecolic acid In cytosol, Cd induce concentration of glycine, ammonium, hydroxyproline and phosphoric acid	[286]
*Brassica napus*	100 µM CdCl_2_; 8 d	Leaves	UPLC/MS	PCA, KEGG	SDMs found as: 644 in sensitive genotype ZD622, and 487 in tolerant genotype CB671 genotype (487) Most of the identified SDMs are involved in the Cd-mediated stress tolerance pathways	[287]

**Table 7:** Role of genetically modified plants in the detoxification and phytoremediation of Cd in soil.

Plant Species	Genes	Phytoremediation Activity	References
*Indian mustard*	*γ*-glutamylcysteine synthetase (*γ*-ECS)	Higher phytochelatins production directly enhanced the Cd phytoremediation capacity of transgenic plants	[291]
*Oryza sativa*	*OsHMA3*	Keep the Cd at roots via sequestrating into root vacuoles	[148]
*Solanum lycopersicum*	*FER*	*FER* lack of function mutant indirectly impaired the Cd translocation from root to aerial parts	[292]
*Arabidopsis*	*NRT1.1*	Controlled the uptake of Cd in roots	[293]
*Arabidopsis*	*AtHMA4*	Better root to shoot translocation of Cd	[294]
*Arabidopsis*	*AtBCC3*	Facilitated the Cd phytoremediation in *Arabidopsis* by enhancing the chelation properties	[145]
*Oryza sativa*	*OsHMA3*	Loss-of-function enhanced root-shoot Cd translocation	[295]
*Arabidopsis*	*AtFC1*	Increased in the accumulation of Cd was observed	[296]
*Arabidopsis*	*AtPDF2.5, AtPDF2.6*	Improved the Cd tolerance accumulation in shoot	[35,290]
*Arabidopsis*	*CAL2*	Hastened the Cd accumulation in shoots	[289]
*Arabidopsis*	*BTS*	*BTS* lack of function mutant enhanced the Cd accumulative characteristics of *Arabidopsis* plants	[248]

**Table 8:** Enlisted studies of arbuscular mycorrhizal fungi (AMF)/fungi and its role in Cd phytoremediation.

Host Plants	AMF/Fungi Species	Specific Function	References
*Salix viminalis*	*Glomus intraradices*	Increased Cd phytoextraction and retained Cd in roots. root and restricted its translocation to shoots	[306]
*Solanum nigrum*	*Paecilomyces lilacinus*	Improved phytoextraction capacity along with enhanced antioxidant systems	[307]
*Tagetes erecta*	*Glomus intraradices, Glomus constrictum* and *Glomus mosseae*	Better phytoextraction of Cd from the soil and also restrict the translocation of Cd from roots to shoots	[308]
*Lpomoea aquatica*	Not specified	Increased the accumulation of Cd along with induced antioxidant enzymes activity and nutrient uptake	[309]
*Cajanus cajan*	*Glomus mosseae*	Amended the generation of phytochelatins and uptake of Cd by roots without accumulating a higher amount of toxic ions	[310]
*Helianthus annuus*	*Rhizophagus irregularis* and *Funneliformis mosseae*	Better phytoextraction and phytostabilization of Cd shown by the sunflower inoculated with AMF	[311]
*Zea mays*	*Glomus intraradices, Glomus constrictum* and *Glomus mosseae*	AMF inoculated plants rapped the Cd in roots and shown improved phytostabilization activity than the control plants	[312]
*Linum usitatissimum*	*Glomus intraradices*	Induced phytoextraction of Cd from the contaminated soil	[313]
*Lonicera japonica*	*Glomus versiforme* and *Rhizophagus intraradices*	AMF inoculated plants showed enhanced phytostabilization capacity of Cd	[314]
*Eichhornia crassipes*	Not specified	Enhanced the Cd accumulation capacity of water hyacinth in Cd contaminated soil and water	[303]
*Solanum nigrum*	Not specified	Boosted the phytoavailability of Cd by lowering the soil pH and altered Cd chemical fractions.	[302]
*Phragmites communis*	*Simplicillium chinense*	Enhanced the Cd phytoremediation by triggering the biosorption process in the host plant	[315]

**Table 9:** List of studies featuring plant growth-promoting bacteria (PGRB) role in Cd phytoremediation.

Host Plants	PGRB Species	Specific Functions	References
*Solanum nigrum*	*Serratia nematodiphila LRE07*	*Serratia nematodiphila LRE07* inoculated plants induced the phytoaccumulation ability of the host plant resulted in to 70% more uptake of Cd	[323]
*Sorghum bicolor*	Bacillus sp. SLS18	Augmented phytoextraction and phytostabilization of Cd at root was observed in the inoculated plants resulted in 65% more uptake of Cd from the contaminated soil	[323]
*Brassica napus*	*Burkholderia* sp. *J62* and *Pseudomonas* *thivervalensis Y-1-3-9*	The inoculated plants showed high level of Cd accumulated in their shots than the control plants	[324]
*Solanum nigrum*	*Pseudomonas* sp. *LK9*	Substantial increase in the Cd uptake of upto 46% was recorded in the host plants inoculated with *Pseudomonas* sp. *LK9*	[325]
*Ocimum ratissimum*	*Arthrobacter* sp.	Cd resistant bacteria along with host plant enhanced the phytoextraction process without altering the quality of grain	[317]
*Eruca sativa*	*Pseudomonas putida ATCC 39,213*	Better phytoextraction resulted in increased Cd uptake (29%) in the inoculated plants than the control	[326]
*Salix dasyclados*	*Streptomyces* sp.	Generation of Siderophores molecules triggered the phytoextraction capacity of the host plant	[327]
*Sedum plumbizincicolaa*	*Endophytic bacterium E6S*	Production of IAA, ACC and organic acid maintained the pH at low level resulted in better phytoextraction of Cd	[328]
*Vetiveria zizanioides*	*Bacillus cereus*	Induced production of phosphate and Siderophores which helps in the phytoremediation of Cd	[329]
